# Fibroblasts and endothelial cells interplay drives hypertrophic scar formation: Insights from in vitro and in vivo models

**DOI:** 10.1002/btm2.10630

**Published:** 2023-12-20

**Authors:** Yaxin Tan, Mengde Zhang, Yi Kong, Fanliang Zhang, Yuzhen Wang, Yuyan Huang, Wei Song, Zhao Li, Linhao Hou, Liting Liang, Xu Guo, Qinghua Liu, Yu Feng, Chao Zhang, Xiaobing Fu, Sha Huang

**Affiliations:** ^1^ College of Graduate Tianjin Medical University Tianjin PR China; ^2^ Research Center for Tissue Repair and Regeneration Affiliated to the Medical Innovation Research Department PLA General Hospital and PLA Medical College Beijing PR China

**Keywords:** cell crosstalk, endothelial cell, fibroblast, scar formation, spheroids

## Abstract

Hypertrophic scar formation is influenced by the intricate interplay between fibroblasts and endothelial cells. In this study, we investigated this relationship using in vitro and in vivo models. Clinical observations revealed distinct morphological changes and increased vascularity at pathological scar sites. Further analysis using OCTA, immunohistochemistry, and immunofluorescence confirmed the involvement of angiogenesis in scar formation. Our indirect co‐culture systems demonstrated that endothelial cells enhance the proliferation and migration of fibroblasts through the secretion of cytokines including VEGF, PDGF, bFGF, and TGF‐β. Additionally, a suspended co‐culture multicellular spheroid model revealed molecular‐level changes associated with extracellular matrix remodeling, cellular behaviors, inflammatory response, and pro‐angiogenic activity. Furthermore, KEGG pathway analysis identified the involvement of TGF‐β, IL‐17, Wnt, Notch, PI3K‐Akt, and MAPK pathways in regulating fibroblasts activity. These findings underscore the critical role of fibroblasts‐endothelial cells crosstalk in scar formation and provide potential targets for therapeutic intervention. Understanding the molecular mechanisms underlying this interplay holds promise for the development of innovative approaches to treat tissue injuries and diseases.

**Translational Impact Statement**

This study investigates the crucial roles and molecular mechanisms underlying the interaction between fibroblasts and endothelial cells during wound healing and scar formation. The findings provide valuable insights into potential therapeutic targets that can be specifically addressed to mitigate scar formation in future investigations. Furthermore, these identified targets can be used for screening small molecule compounds with clinical applications, thereby offering pathways to develop more effective preventive and therapeutic strategies.

## 1 INTRODUCTION

Scar formation is a dynamic and interactive process involving multiple cell types, including fibroblasts and endothelial cells.^1^, ^2^, ^3^ Fibroblasts play a crucial role in the formation of scars and fibrosis, and therefore strategies aimed at improving wound healing and reducing fibrosis often target the function and activation of fibroblasts.^4^, ^5^, ^6^ Following injury, fibroblasts are recruited by platelets and inflammatory cells to the damaged site where they begin synthesizing extracellular matrix (ECM) molecules including collagen.^7^ This process is known as fibroblast activation.^8^, ^9^ Over time, fibroblasts become the dominant cell population at the wound site and their activity drives the scarring process.^10^ Therefore, fibroblast activation is a crucial step in scar formation, with various activation modes, such as TGF‐β/Smad signaling pathways, YAP/TAZ signaling pathways, and IL‐6 signaling pathways, which induce fibroblasts migration, proliferation, and transformation, promoting scar formation.^11^, ^12^, ^13^

Endothelial cells are also crucial factors that affect tissue repair and scar formation.^14^, ^15^, ^16^ Early in the wound healing process, endothelial cells are activated, releasing “vascular secretory factors”, including vascular endothelial growth factor (VEGF), platelet derived growth factor (PDGF), and basic fibroblast growth factor (bFGF), among others.^17^, ^18^, ^19^ These factors activate surrounding cells, including fibroblasts, macrophages, and epithelial cells, accelerating the wound healing process.^20^, ^21^ Additionally, endothelial cells depend on ECM molecules such as fibronectin to interact with fibroblasts, promoting their activation and proliferation, and further promoting scar formation.^22^, ^23^

Although the mechanism of fibroblasts and endothelial cells in scar formation has been clarified, the interaction mechanism between fibroblasts and endothelial cells and endothelial‐derived efficacy on fibroblast activation in scar formation is still unclear. We hypothesize that the interaction between fibroblasts and endothelial cells in scars is mainly achieved through the following ways: (1) Endothelial cells can also activation of fibroblasts and affect the process of scar formation.^24^ (2) Interaction through secretion of factors. Fibroblasts and endothelial cells interact by secreting factors that promote their mutual behavior directly or indirectly, thus further promoting scar formation.^25^, ^26^ (3) Fibroblasts and endothelial cells can form dynamic signaling pathways through tight cellular connections, regulating important events such as cell cycle regulation and cell apoptosis.^27^, ^28^

In this study, we aimed to investigate the interaction between fibroblasts and endothelial cells in scar formation from in vitro and in vivo models. To verify our hypothesis, we first compared blood vessels from proliferative scars with those from adjacent normal skin using histological methods. We then explored the regulatory effects of vascular secretory factors on fibroblasts behavior using enzyme‐linked immunosorbent assay (ELISA). To simulate these interactions in vivo, we established a multicellular spheroids model containing human endothelial cells and fibroblasts and employed high‐throughput sequencing to identify related genes and signaling pathways. Compared to cells cultured in 2D petri‐dish, cells in 3D cell‐spheres exhibit improved biological characteristics and have tighter cell contaction.^29^ Besides, 3D multicellular spheres can better mimic the complex morphology and heterotypic cell interactions found in normal tissues.^30^ Using single‐cell sequencing dataset, we clarified the molecular interactions between endothelial cells and fibroblasts in their resident microenvironment. Our findings not only highlight the critical role of endothelial cells and fibroblasts in scar formation but also offer new avenues for developing potential therapeutic strategies for treating fibrosis and scarring.

## 2 MATERIALS AND METHODS

### 2.1 Human subjects

This study was approved by the Medical Ethics Committee of the Chinese PLA General Hospital in accordance with the Declaration of Helsinki principles, and written informed consent was obtained from each patient (S2020‐457‐01). The inclusion criteria were: (a) Patients who are willing to undergo hypertrophic scar resection surgery and/or autologous skin transplantation surgery. (b) Patients with clinically typical hypertrophic scars caused by wounds or burns: the lesion area appears red or brown with harder texture and protrudes from normal skin with clear boundaries. (c) Hypertrophic scars with typical pathological features (significantly thickened dermis, few skin appendages). The exclusion criteria were: (a) Patients with serious life‐threatening diseases. (b) Patients with skin diseases that affect skin wound healing. (c) Patients who cannot cooperate with the operation or examination. (d) Patients with hypertrophic scars formed after resection of malignant tumors. (e) The course of hypertrophic scars is less than 6 months. Hypertrophic scar and normal skin tissues of 13 patients (6 female and 7 male patients; age range: 3–33 years) were collected from the Department of Surgery in our hospital. All patients met criteria for inclusion and exclusion.

### 2.2 Cell isolation and culture

We collected scar tissues within 6 h of surgery and soaked it in phosphate buffer saline (PBS) with 2% penicillin/streptomycin (P/S) for 5 min. The epidermis and hypodermis were mechanically removed from the scar tissue with a scalpel. The remaining dermis was minced into 1 mm^3^ pieces in sterile culture dishes. These pieces were then transferred to culture dishes precoated with fetal bovine serum (FBS, Gibco) and incubated in complete Dulbecco's modified Eagle's medium (DMEM, Gibco) for 7 days in a humidified atmosphere at 37°C, 5% CO_2_ until the cells reached 70%–80% confluence.^31^ Passages 5–15 were used for subsequent experiments and named as scar‐derived fibroblasts (SCF).

Human skin fibroblast cell line (HSF, iCell) was cultured under 37°C in 5% CO_2_ conditions in complete medium (DMEM + 10% FBS + 2% P/S). Passages 5–15 were used for the following experiment.

Human umbilical vein endothelial cells (EC, iCell) were cultured in endothelial cell medium (EDCM, Sciencell) at 37°C, 5% CO_2_. Passages 2–8 were used for the following experiment.

### 2.3 OCTA imaging

Optical coherence tomography angiography (OCTA) images were acquired using a fiber‐based spectral‐domain OCTA system. The light source was a broadband super‐luminescent diode with a central wavelength at 1325 nm and a full‐width‐half‐maximum bandwidth of 100 nm. This instrument enables imaging of the cutaneous microvasculature with a 20 kHz line‐scan rate and an imaging resolution of 15 and 20 μm in the axial and transverse directions, respectively. The scanning range used in this study was 3 × 3 mm^2^ (*x* × *y*) and the total acquisition time for one field was about 5 s. The total power was in the range of 5–10 mW. After cleansing of the sampling site, OCTA images were captured using a hand‐held probe at the scar site, the adjacent normal site, and the intersection of these two sites.

### 2.4 Histology, immunofluorescence, and immunocytochemistry

Skin and scar tissues were fixed in 4% paraformaldehyde and embedded in paraffin according to standard procedures. Hematoxylin and eosin (H&E) staining was performed to visualize the anatomical structure of the samples.

For immunofluorescence, antigen retrieval and blocking were performed, and sections were incubated with primary antibodies (Ki67 [ab16667, Abcam], Col1A1 [ab138492, Abcam], CD31 [66065‐2‐Ig, Proteintech], and α‐SMA [ab32575, Abcam]). Then, sections were incubated with secondary antibodies (CoraLite488‐conjugated Goat Anti‐Rabbit IgG [SA00013‐2, Proteintech] or CoraLite594‐conjugated Goat Anti‐Mouse IgG [SA00013‐3, Proteintech]) at room temperature. Finally, 4′,6‐diamidino‐2‐phenylindole (DAPI) Fluoromount‐G (0100‐20, SouthernBiotech) was used to stain nucleus. Images were captured with a confocal microscope (Leica, SP8 FALCON).^32^

For immunocytochemistry, antigen retrieval and blocking were performed, and sections were incubated with primary antibodies (CD31 [ab281583, Abcam]) for 18 h at 4°C. Then, sections were incubated with secondary antibody biotinylated goat anti‐rabbit immunoglobulins followed by counterstaining with Mayer's hematoxylin.

### 2.5 Condition medium

The original medium was discarded after cell fusion of HSF, SCF, or EC cells reached 90%. Cells were washed with PBS three times, and 10 mL serum‐free mixed media (DMEM:EDCM = 1:1) was added. It is worth noting that in order to exclude the influence of growth factors added to EDCM, we chose to omit the use of these factors (ECGS), relying solely on a conventional serum‐supplemented cell culture medium to support the cultures. Then, cells were cultured for another 48 h, after which the medium was collected. The medium was centrifuged at 3000*g* for 5 min and supernatant as conditioned medium (CM) was stored at −80°C for consequent tests.

### 2.6 Cell viability assay

Cell viability was measured with cell counting kit‐8 (CCK‐8; Dojindo) assay. Cells were seeded in 96‐well plates at a density of 3 × 10^3^ cells/well and treated as indicated. After incubation with CCK‐8 reagent for 2 h at 37°C, cell proliferation was assessed by measuring absorbance at 450 nm (OD_450_) using a SPARK 10 M plate reader (TECAN). The sampling time points were 0, 24, 48, 72, and 96 h.

### 2.7 Cell migration assay

We evaluated the migration ability of fibroblasts and endothelial cells using the wound scratch assay and transwell assay.^33^ For the wound scratch assay, cells were seeded in 6‐well plates at a density of 2 × 10^6^ cells/well. When reaching 90% confluence, the cells were scratched perpendicularly using a pipette tip, and washed twice with PBS. The medium was changed to mixed media (ECCM and the mixed media without cultured cells) with no FBS. The closure of the scratches was documented using optical microscopy. For transwell assay, we placed 2 × 10^5^ cells in the lower compartment of 6.5‐mm Transwells containing 8.0‐μm‐pore inserts (Corning), and cultured them for 5 h. Then, we resuspended another set of cells in mixed medium (DMEM:EDCM = 1:1, EDCM without the addition of ECGS) supplemented with 2% FBS and seeded them into the upper compartments at a density of 5 × 10^4^ cells/well. After coculturing for another 18 h, the cells were fixed with 4% paraformaldehyde. Non‐migrated cells were removed with a cotton swab, and the migrated fibroblasts were stained with 0.1% crystal violet (Solarbio) for 30 min and rinsed them three times with PBS. The number of migrated cells was quantified by counting at least nine randomly selected fields in three replicates.

### 2.8 ELISA

ELISA was performed according to the manufacturer's protocol of the ELISA kit (Meimian). Briefly, the cell culture medium was centrifuged at 4°C for 20 min (3000 rpm) and the supernatant was collected. Next, the supernatant and HRP‐conjugate reagent were added to the microelisa stripplate respectively. After incubation at 37°C for 30 min, the liquid was discarded and the plates were thoroughly washed. Next, chromogen solution was added to each well for 10 min at 37°C, protection from light. Finally, stop solution was added to each well, and the absorbance was measured at 450 nm. Each sample was assessed at least three replicates.

### 2.9 Spheroid fabrication

We fabricated fibroblast‐only or multicellular spheroids in 96‐well plates with an ultra‐low attachment surface (Labselect, China). A volume of 150 μL of cell suspension was pipetted into each well. For co‐cultured spheroids composed of fibroblasts and endothelial cells, cell suspensions of each cell type were prepared, and the cells were counted prior to mixing them at a 1:1 ratio. Briefly, 2 × 10^5^ cells/mL was prepared for fibroblasts and endothelial cells suspensions, respectively. A 150 μL cell mixture of these two cell types was prepared in accordance with the 1:1 ratio (fibroblasts and endothelial cell 1:1 = 75:75 μL). The plates were incubated under 37°C with 5% CO_2_ conditions for 36 h, and the spheroids were subsequently removed for further experiments.

Spheroids were fixed in 4% paraformaldehyde and embedded in optimal cutting temperature compound (Sakura) according to standard procedures. Frozen slices of spheroids were prepared via Leica frozen slicer with a thickness of 10 μm.^30^

### 2.10 Live/dead assay analysis

To assess cell viability in spheroids, we used live/dead assay kits (L3224, Invitrogen) to distinguish live cells (green fluorescence) from dead cells (red fluorescence) through observation using a confocal microscope (Leica, SP8 FALCON).

### 2.11 RNA‐sequencing

We performed mRNA extraction from isolated cell protrusions using the fixed and recovered intact RNA protocol. Briefly, the cell spheroids were dissociated into single‐cell suspensions using Trypsin–EDTA solution (Solarbio), and CD31^−^ fibroblasts were sorted using fluorescence‐activated cell sorting (FACS). RNA was isolated using TRLzol reagent (Ambion), and sequencing was performed using Illumina NovaSeqTM 6000 by LC Sciences. Genes with a false discovery rate (FDR) parameter less than 0.05 and an absolute fold change ≥2 were considered differentially expressed genes (DEGs), which were then subjected to enrichment analysis of Gene Ontology (GO) functions and KEGG pathways. All DEGs were mapped to GO terms in the Gene Ontology database (http://www.geneontology.org/), gene numbers were calculated for every term, and significantly enriched GO terms in DEGs compared to the genome background were defined by hypergeometric test. KEGG is the major public pathway‐related database. Pathway enrichment analysis identified significantly enriched metabolic pathways or signal transduction pathways in DEGs compared with the whole genome background.

### 2.12 Sample preparation and tissue dissociation

The skin tissues were dissociated into single‐cell suspensions using mechanical cutting and enzymatic digestion according to the manufacturer's instructions of 10× Genomics Chromium Single‐Cell 3′kit (V3). The overall cell viability was confirmed by trypan blue exclusion, which needed to be above 85%. Single‐cell suspensions were counted using a hemocytometer, and the concentration was adjusted to 700–1200 cells/μL.

### 2.13 Library preparation and RNA sequencing

Single‐cell suspensions were loaded to 10× chromium to capture single cells. The following cDNA amplification and library construction steps were performed according to standard protocols. Libraries were sequenced on an Illumina NextSeq 6000 sequencing system (double‐end sequencing, 150 bp) by LC‐Bio Technology Co., Ltd.

### 2.14 Single‐cell RNA sequence data analysis

Cell Ranger was used to align the raw data with the human reference genome (hg19), perform data quality assessment, integrate multiple sample data, normalize gene expression levels, and construct a gene expression matrix. Further data filtering resulted in 50,941 cells passing the quality control threshold. Principal component analysis (PCA) and Uniform Manifold Approximation and Projection (UMAP) were employed to visualize the data. Cell clusters were then visualized and identified using the Cell Marker database and published literature. In total, 14 cell types were identified for subsequent analysis.

To assess cell–cell communication molecules between different cell types, we used CellPhoneDB software to infer the intercellular communication network from single‐cell transcriptome data. Only receptors and ligands expressed in more than 10% of the cells in the specific cell types were considered in the analysis. The upregulated ligand and acceptor were mapped to GO terms in the Gene Ontology database. GO enrichment analyses were performed using Metascape (https://metascape.org).

### 2.15 Statistical analysis

All values are presented as the means ± standard deviation (SD) calculated from the average of at least three biological replicates. The one‐way analysis of variance with a post hoc Bonferroni's test and unpair *t*‐test was used to analyze the results using GraphPad Prism 8.0 (*p < 0.1, **p < 0.01, ***p < 0.001, and ****p < 0.0001).

## 3 RESULTS

### 3.1 Increased density of blood vessels in pathological scars

**FIGURE 1:**
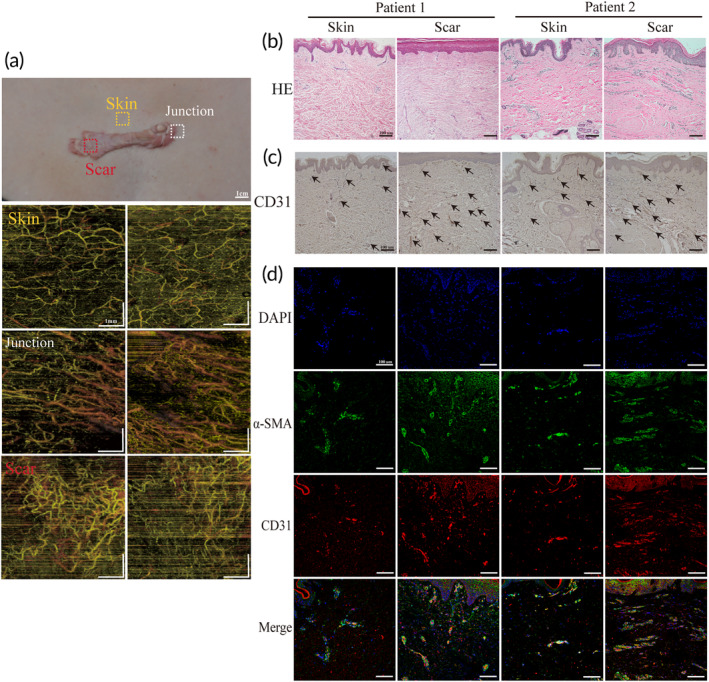
Assessment of microvessels in pathologic scar of human skin using OCTA and histological section. (a) OCTA images in pathologic scar, normal skin adjacent to the scar, and their junction site. (b) H&E staining of pathologic scar and normal skin (scale bar: 100 μm). (c) Immunohistochemistry staining of CD31 (scale bar: 100 μm). (d) Dual‐labeled immunofluorescence staining of CD31 and α‐SMA (scale bar: 100 μm).

Scar formation has been reported to be associated with uncontrolled angiogenesis or the delayed regression of blood vessels.^2^ We used OCTA and histological sectioning to examine the morphology and number of blood vessels in human scar versus normal skin samples. First, we used OCTA to examine three areas of the patient's skin: the scar site, the normal site adjacent to the scar, and the intersection of these two sites. At the scar site, blood vessels exhibited a composite, curved morphology, including comma‐, cup‐, and spiral‐shaped structures, with obvious dilatation and a reticular arrangement of the vascular structure. In contrast, the vessels beneath the normal epidermis adjacent to the scar had a finer diameter and a uniform vascular distribution with a linear bifurcated morphology. At the junction between the two sites, a combination of the aforementioned two characteristic vessels was observed (Figure 1a). Then, the pathological scar and normal skin were assessed by H&E staining. The scar tissue is initially observed as a thickened epidermis. Additionally, the number of nuclei in the dermis was markedly raised, cord‐like structures were enriched, and collagen bundles were coarse and dense with disordered deposition (Figure 1b). We then used immunohistochemical staining to detect blood vessels, and the results demonstrated that CD31‐positive endothelial cells (brown) were significantly increased in the scar samples (Figure 1c). Finally, we used CD31 and α‐SMA dual‐labeled immunofluorescence co‐localization to locate the vessels. Immunofluorescence revealed the red marker (CD31), surrounded by the green marker (α‐SMA), which is consistent with the anatomical location of endothelial cells and perivascular smooth muscle cells. Compared to normal skin, the scar region displayed a dense network of blood vessels, consistent with immunohistochemistry findings (Figure 1d). In summary, we observed a significant increase in the distribution of blood vessels within human scar samples compare with normal skin.

### 3.2 Fibroblasts and endothelial cells interaction via cytokines

**FIGURE 2:**
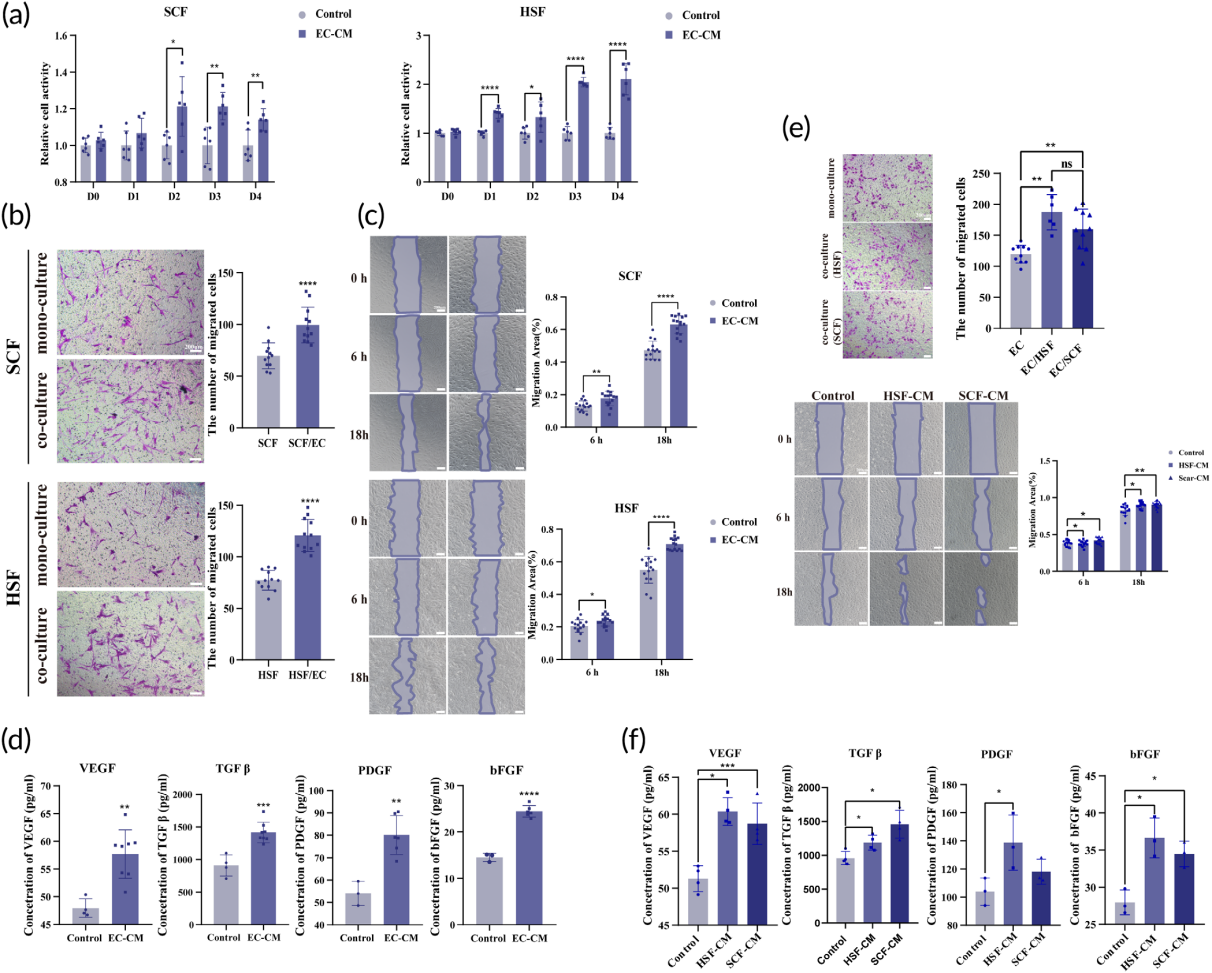
The interaction between fibroblasts and endothelial cells in indirect coculture system. (a) CCK8 assay was performed to detect the viability of fibroblast (*n* = 6). (b) Transwell (*n* = 12) was performed to detect the migration of fibroblast. (c) Scratch assays (*n* = 15) were performed to detect the migration of fibroblast. (d) ELISA results show that the levels of VEGF, TGFβ, PDGF, and bFGF in EC‐CM. (e) Scratch assays (*n* = 15) and Transwell (*n* ≥ 6) were performed to detect the migration of endothelial cells. (f) ELISA results show that the levels of VEGF, TGFβ, PDGF, and bFGF in SCF‐CM and HSF‐CM (*p < 0.1, **p < 0.01, ***p < 0.001, and ****p < 0.0001 compared with control group; scale bar: 200 μm).

To determine whether endothelial cells would regulate the biological behavior of fibroblasts through the paracrine pathway, we used two indirect co‐culture systems (conditioned medium and transwell). Initially, we investigated the effect of endothelial cells on the proliferation of HSF and SCF. CCK8 assay demonstrated that endothelial cell‐conditioned medium (EC‐CM) significantly promoted the proliferation of SCF and HSF (Figure 2a). Subsequently, we examined the effect of endothelial cells on the migratory capacity of SCF and HSF. Transwell assays showed that more fibroblasts migrated in the co‐culture group (Figure 2b). Scratching experiments indicated that fibroblasts in the EC‐CM group exhibited significantly higher migration speeds (Figure 2c). For EC‐CM, we used DMEM and EDCM without the addition of endothelial cell growth supplements (ECGS). The rationale for this was to reduce masking and interference of the potential growth factors‐mediated crosstalk to cell behavior. Furthermore, ELISA was applied to quantify the levels of classic fibroblast activating factors in the EC‐CM. Compared to the control group, the levels of VEGF, TGF‐β, PDGF, and bFGF were significantly higher in the EC‐CM group (Figure 2d).

To explore the potential of fibroblasts to modulate endothelial cells migration via the paracrine pathway, two indirect co‐culture systems have been established as previously described. Transwell assays revealed that the inclusion of either SCF or HSF in the lower chamber can result in a significant increase in the number of migrating endothelial cells (Figure 2e). Moreover, scratching experiments indicated that culturing endothelial cells with either scar derived fibroblasts‐conditioned medium (SCF‐CM) or human skin fibroblasts‐conditioned medium (HSF‐CM) significantly increased their migration ability (Figure 2e). Additionally, using ELISA, we quantified the levels of classical pro‐angiogenic factors within SCF‐CM and HSF‐CM, and our results demonstrated significantly elevated levels of VEGF, TGF β, and bFGF compared to the control group (Figure 2f).

### 3.3 Construction of a stable co‐culture multicellular spheroid model

**FIGURE 3:**
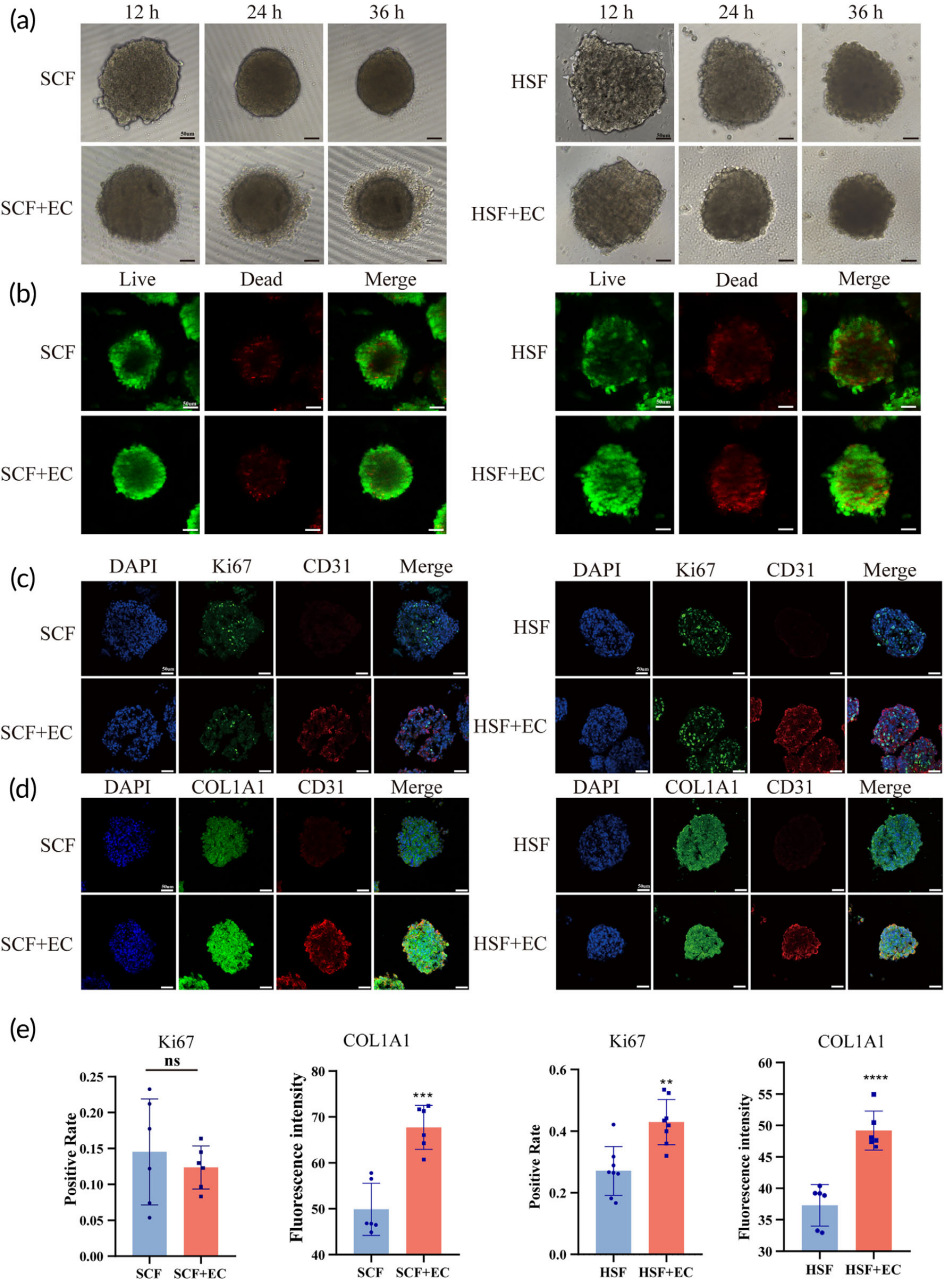
Properties and characteristics of multicellular spheroid model. (a) The spheroids' morphological changes under the light microscope. (b) The live/dead test showed a good cell viability of the model. (c,d) Immunofluorescence imaging of Ki67 and COL1A1 in spheroids after being cultured for 36 h in vitro. (e) Quantification of Ki67 and COL1A1 expression level (*n* ≥ 6) (**p < 0.01, ***p < 0.001, and ****p < 0.0001; scale bar: 50 μm).

3D suspension culture is the optimal approach to simulate cell–cell and cell–ECM interactions in vivo.^34^, ^35^ In this research, we generated two types of human‐derived multicellular spheroids, specifically SCF + EC co‐culture spheroids and HSF + EC co‐culture spheroids, using suspension culture. Fibroblast mono‐culture spheroids (SCF mono‐culture cell spheroids, HSF mono‐culture cell spheroids) were utilized as control groups. To assess the stability of the model, we characterized the cell spheroids in terms of four criteria: sphericity, cell viability, cell proliferation, and fibroblast collagen secretion. Firstly, light microscopic observations indicated that cells gradually aggregated and integrated into well‐defined spherical structures that persisted throughout the culture period (Figure 3a). Subsequently, live/dead analysis demonstrated predominantly live cells (green) within the cell spheroids, indicating robust cell survival during suspension culture (Figure 3b). We then evaluated the cell proliferation of the spheroids through Ki67 staining. The results indicated that both SCF and SCF + EC co‐cultured cell spheroids retained proliferative activity without any significant statistical difference (Figure 3c,e). HSF + EC co‐cultured cell spheroids exhibited higher level of proliferative activity compared to mono‐cultured HSF cell spheroids (Figure 3c,e). Finally, we labeled the cell spheroids with collagen‐associated markers (COL1A1) to visualize fibroblast collagen secretion. The results indicated that co‐cultured multicellular spheroids exhibited more intense collagen secretion than mono‐culture cell spheroids (Figure 3d,e). In summary, we constructed multicellular spheroids showed exceptional spheroid‐forming ability, favorable cell viability, proliferative capacity, and fibroblast function.

### 3.4 Transcriptomic changes occur in fibroblasts spheroids co‐cultured with endothelial cells

**FIGURE 4:**
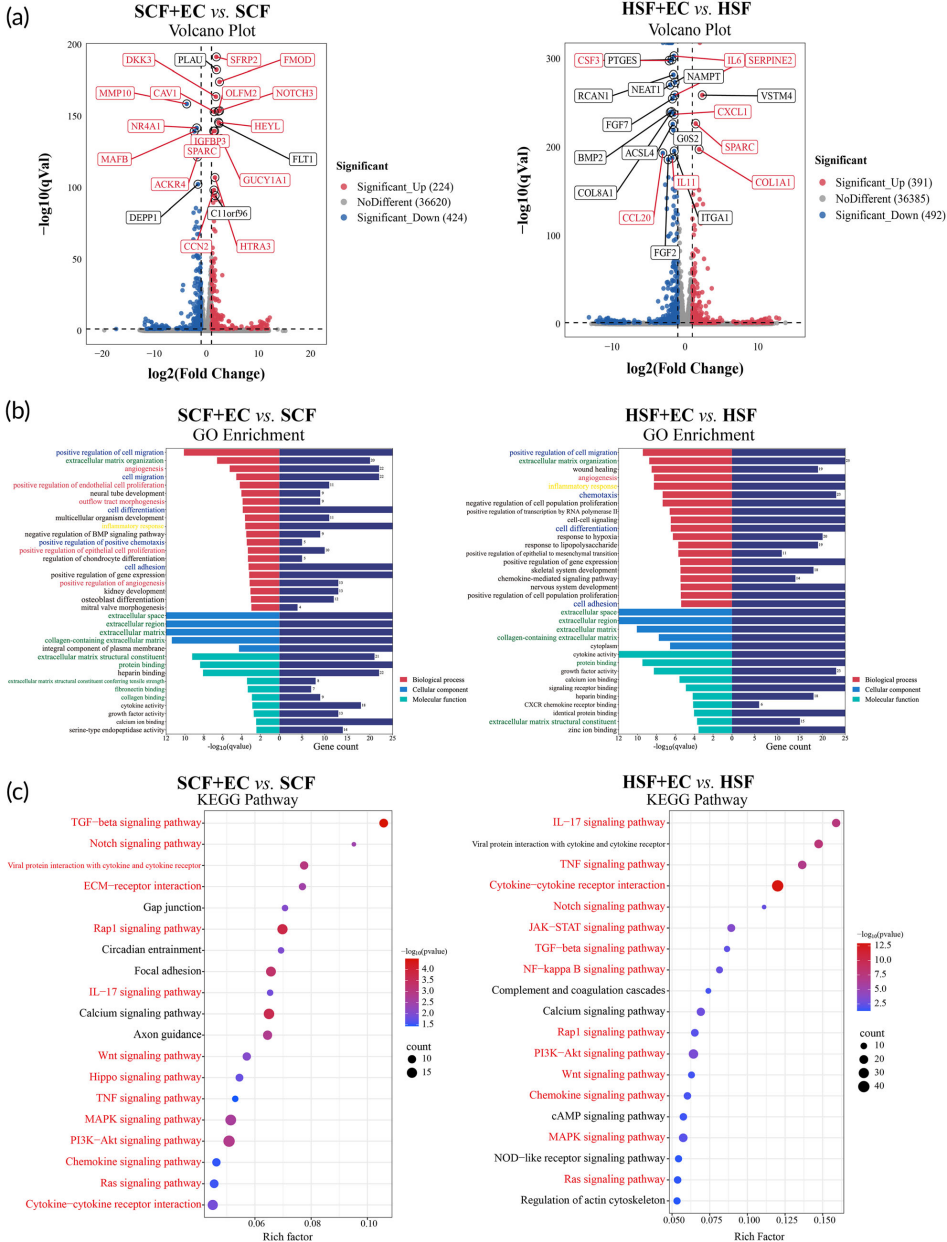
Differential expression genes analysis of fibroblast in co‐culture multicellular spheroids compared to monoculture cell spheroids. (a) Volcano plot of DEGs. (b) GO analysis showed these differentially expressed genes were enriched in extracellular matrix (green font), angiogenesis (red font), cell behavior‐related (blue font), and inflammatory‐related (yellow font) related GO terms. (c) Enrichment plots of KEGG pathway for representative signaling pathways using the DEGs.

The suspension co‐culture multicellular spheroid system we constructed exhibits partial resemblance to the process of human wound healing and scar formation. To systematically investigate the interaction between fibroblasts and endothelial cells within the co‐culture multicellular spheroid system, we compared the transcriptional gene expression patterns between fibroblasts in co‐culture and mono‐culture cell spheroids. Firstly, in the “SCF + EC versus SCF” comparison, a total of 37,268 genes were sequenced, revealing 224 up‐regulated genes and 424 down‐regulated genes with |log2 fold change| >1, *p* < 0.05 (Figure 4a). Similarly, in the “HSF+EC versus HSF” comparison, 37,268 genes were sequenced in total, with 391 up‐regulated and 492 down‐regulated genes meeting the aforementioned criteria (Figure 4a). Co‐culturing fibroblasts with endothelial cells resulted in significant changes in gene expression compared to mono‐cultured fibroblasts. Subsequently, we conducted GO enrichment analysis for the differentially expressed genes to investigate the potential impact of endothelial cells on fibroblasts. Notably, the GO terms related to the extracellular matrix, such as “extracellular matrix organization”, “extracellular space”, and “extracellular matrix structural constituent”, were enriched in “SCF + EC versus SCF” and “HSF + EC versus HSF”, which is consistent with the protein characterization of spheroids mentioned above (green font, Figure 4b). In addition, the following terms were enriched in “SCF + EC versus SCF” and “HSF + EC versus HSF”: the angiogenesis‐related GO terms, such as “angiogenesis”, “positive regulation of angiogenesis” (red font, Figure 4b); cell behavior‐related GO terms, such as “positive regulation of cell migration”, “cell differentiation”, and “cell adhesion”(blue font, Figure 4b); inflammatory‐related GO term, such as “inflammatory response” (yellow font, Figure 4b). Finally, KEGG pathway enrichment analysis showed that the differentially expressed genes in the suspension multicellular co‐culture system were enriched classical fibrosis signaling pathways, including TGFβ, Notch, IL17, Wnt, MAPK, and PI3K‐AKT signaling pathway (red font, Figure 4c).

### 3.5 Signaling communication between fibroblasts and endothelial cells

**FIGURE 5:**
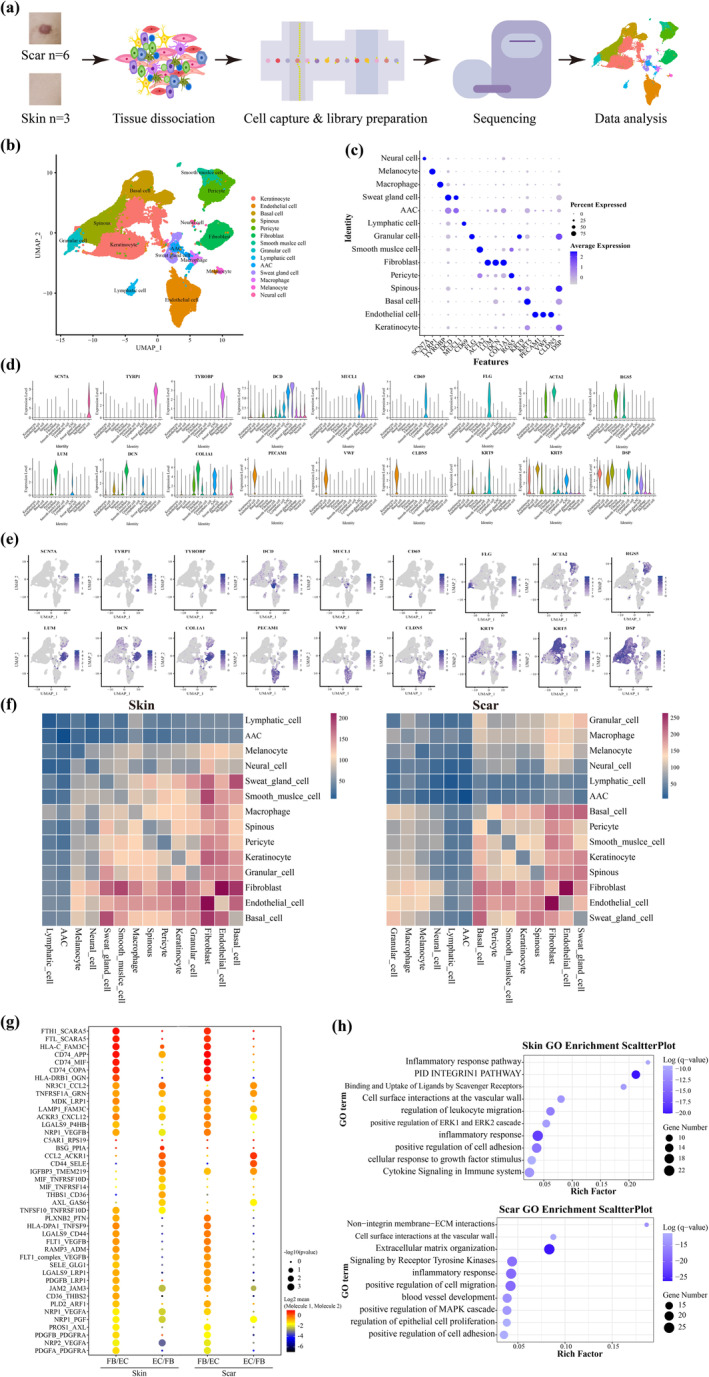
Cell–cell communications between fibroblasts and endothelial cells in scars and relatively normal skin tissues. (a) Workflow. Samples were harvested from nine individuals for scRNA‐seq. (b) UMAP plot of 50,941 human cells, colored by cluster. (c)–(e) Bubble Plot, Violin plot, and UMAP demonstrate the expression level and distribution of specific genes in different cell types. (f) Heatmap depicting interactions among all cell types obtained with CellPhoneDB. (g) Dot plot showing the representative ligand–receptor pairs between fibroblast and endothelial cells in normal skin versus scar. The dot size represents the *p* value. The dot color represents the mean of the average expression level of the two interacting molecules in their respective clusters. (h) Scatter plot displaying the upregulated receptor and ligand genes by GO enrichment analysis. The rich factor indicates the ratio of the number of genes with significant differences annotated as a specific GO term to all genes annotated as the same GO term. Dot size represents the number of genes with a significant difference enriched in a specific GO term. The dot color represents the *p* value obtained by GO analysis. *p* < 0.05 was used to indicate significant enrichment.

To investigate the cellular interaction between various cell types in hypertrophic scar samples and normal skin samples, we obtained nine skin samples from six patients with hypertrophic scars and three healthy controls. scRNA‐seq was performed on the dissociated cells from these samples (Figure 5a). After quality control, the transcriptomes of 50,941 cells were obtained. The filtered data were then integrated, dimensionally reduced, and clustered in an unsupervised method. In this analysis, combined with the expression of signature genes for each cluster, 14 cell types were identified (Figure 5b). These cell types include fibroblasts (marked by LUM, DCN, and COL1A) and endothelial cells (marked by vWF, PECAM, and CLDN)^36^, ^37^ (Figure 5c–e). To explore the interplay between fibroblasts and endothelial cells in their resident microenvironment, we revealed the cell–cell communication networks based on CellPhoneDB.^38^ Heatmaps shows that close intercellular communication between fibroblasts and endothelial cells in both normal and diseased states, with particularly pronounced interactions observed in scar tissue (Figure 5f). Then, we visualized the receptor–ligand pairs between fibroblasts and endothelial cells using a bubble diagram (Figure 5g). To further elucidate the biological functions of fibroblast–endothelial communication, we performed GO analysis on the ligand–receptor genes identified in the enriched CellPhoneDB results. These ligand–receptor genes were primarily associated with extracellular matrix organization, cell migration, cell adhesion, cell chemotaxis, blood vessel development, and inflammatory response (Figure 5h).

## 4 DISCUSSION

Clinical observations have consistently indicated that the pathological scar site exhibits a heightened level of redness compared to the surrounding normal tissue.^39^, ^40^ The underlying mechanism responsible for this phenomenon in scar formation remains poorly understood. To address the knowledge gap, we initially employed OCTA to investigate the in vivo blood vessel profiles within the scar area as well as the adjacent unaffected area.^41^ Our data revealed an increase in the number of blood vessels along with distinct morphological changes exclusively at the scar site (Figure 1a). These findings were corroborated through further validation employing immunohistochemistry and immunofluorescence techniques, which consistently yielded similar results (Figure 1b). Hence, it is imperative to explore the intricate relationship between scar formation and angiogenesis. Current research indicates that scar formation is a multifaceted process involving various cell populations, prominently fibroblasts and endothelial cells.^3^ In pathological scarring, fibroblasts assume a dominant role, while endothelial cells are primarily responsible for vascular composition.^5^, ^42^ Here, we provide insights into the interplay between these two cell types, shedding light on their contribution to scar formation and the associated increased vascularity.

The interplay between fibroblasts and endothelial cells drives hypertrophic scar formation by influencing the local microenvironment.^43^ Initially, we hypothesized that these changes are mediated through paracrine signaling. To explore the effect of endothelial cells on fibroblasts, we established two indirect co‐culture models. The experimental results revealed that endothelial cells enhance both the cellular proliferation and migration of fibroblasts (Figure 2a,b). Moreover, the levels of VEGF, PDGF, bFGF, and TGF‐β were significantly higher in the conditioned medium from endothelial cells than in the medium without cultured endothelial cells (Figure 2c). Previous studies have linked the proliferation and migration of fibroblasts to signaling pathways involving PDGF, bFGF, and TGF‐β. Notably, VEGF, PDGF, bFGF, and TGF‐β serve as activators of these pathways.^44^, ^45^, ^46^ These findings indicate that endothelial cells modulate fibroblast behavior by secreting cytokines that alter the local microenvironment. Additionally, we investigated whether fibroblasts exert a reciprocal effect on endothelial cells migration through paracrine secretion. Research suggested that the formation of tip cells is crucial for angiogenesis. These specialized cells act as guides, directing other endothelial stem cells to migrate, and their development is intricately connected to the VEGF/Notch pathway.^47^, ^48^ Moreover, VEGF, TGF‐β, and bFGF have been implicated in angiogenesis.^49^ Therefore, fibroblasts and endothelial cells mutually regulate each other's behavior within the local microenvironment by releasing cytokines, thereby promoting wound healing and scar formation.

To facilitate a more accurate representation of cell–cell interactions, we constructed a suspended co‐culture multicellular spheroid model. Based on the literature, we selected cell spheroids with a diameter of approximately 200 μm to induce moderate hypoxia due to restricted oxygen diffusion.^50^ Notably, the cell spheroids co‐cultured with macrophage cells and fibroblasts exhibited the highest expression levels of fibrosis‐related genes.^27^ Live/death experiments demonstrated satisfactory cell viability within the constructed cell spheroids, while Ki67 staining indicated the retained proliferative capacity of cells (Figure 3b,c). However, the scar‐derived fibroblasts proliferation capacity did not significantly differ between co‐cultured and mono‐cultured cell spheroids. Further investigations are required to elucidate the underlying reasons for this observation. COL1A1 staining revealed normal expression of the fibrotic phenotype within the cell spheroids (Figure 3d). Moving towards a molecular‐level assessment, we sequenced the fibroblasts within the constructed cell spheroids, using suspended monoculture fibroblast spheroids as the control. The sequencing analysis highlighted differentially expressed genes associated predominantly with four GO terms: extracellular matrix, cellular behaviors, inflammatory response, and pro‐angiogenic activity (Figure 4b). These GO term enrichments align with single‐cell sequencing findings investigating closely interacting receptor–ligand pairs (Figure 5h), which are crucial for wound healing and scar formation. We conducted KEGG pathway enrichment analysis on the differentially expressed genes to explore the pathways involved in endothelial cell‐mediated regulation of fibroblasts. Results emphasized that endothelial cells regulate fibroblast activity through multiple signaling pathways, including TGF‐β, IL‐17, Wnt, Notch, PI3K‐Akt, and MAPK pathways (Figure 4c). The TGF‐β signaling pathway exerts its influence on fibroblast activity through both canonical (Smad‐dependent) and non‐canonical pathways, such as PI3K‐AKT, Erk, and P38‐MAPK, leading to fibroblast transformation, collagen deposition, and proliferation.^51^, ^52^ In the context of liver fibrosis, IL‐17 amplifies the response of hepatic stellate cells to TGF‐β by enhancing the production of inflammatory chemokines, ultimately contributing to increased fibrosis.^53^ Additionally, in systemic sclerosis, IL‐17 promotes the proliferation of skin fibroblasts.^54^ The Wnt signaling pathway triggers the activation and expansion of endocardium‐derived fibroblasts during cardiac remodeling.^55^ Notch3, acting as a vital receptor on fibroblasts, modulates fibroblast differentiation and expansion. It is important to note that much of the Notch signaling activation occurs through direct contact between ligand and receptor cells.^56^

Notably, both our sequencing results establish a connection between fibroblast–endothelial cell communication and inflammation. GO analysis of co‐cultured cell spheroids revealed enrichment related to the inflammatory response, while KEGG analysis demonstrated enrichment in IL‐17, TNF, and Wnt pathways (Figure 4b,c). Similarly, GO analysis from single‐cell sequencing identified enrichment in genes associated with the inflammatory response and regulation of leukocyte migration (Figure 5h). These findings indicate that communication between fibroblasts and endothelial cells plays a pivotal role in coordinating the inflammatory response. Existing research suggests that endothelial cells contribute to tissue fibrosis by either initiating a direct inflammatory response or modulating an already established inflammatory process.^57^ For example, in systemic sclerosis, endothelial cell injury triggers the enhanced secretion of inflammatory factors, recruitment of immune cells, and subsequent fibroblast activation, excessive collagen synthesis, and fibrosis.^58^ Similarly, in pulmonary fibrosis, endothelial cells secrete pro‐fibrotic mediators that stimulate fibroblast activation, proliferation, and collagen synthesis.^59^ Fibroblasts, in turn, secrete cytokines, chemokines, and other inflammatory factors that further enhance the inflammatory response. In conclusion, the presence of an inflammatory environment significantly promotes fibrosis. Studying the relationship between the inflammatory response and fibroblast–endothelial cell crosstalk may pave the way for new directions in our future research.

Various research methods have been employed to investigate the relationship between scar formation and angiogenesis, each with its own advantages and limitations. In terms of histological analysis, we utilized OCTA, a non‐invasive imaging technique similar to ultrasonography, commonly used for visualizing ocular fundus vasculature. This technique allows us to examine blood vessels beneath the scar, providing real‐time visualization in vivo.^35^ Utilizing OCTA overcomes drawbacks associated with conventional histology, such as time‐consuming procedures, sample preservation requirements, and the risk of worsening existing scarring through secondary excision.^60^

To study the paracrine role of cells, we employed an indirect co‐culture system that physically isolates the cells, allowing only the passage of cytokines. While this approach enables us to identify the source of cytokines and observe target cell behavior, it does impede direct cell‐to‐cell interactions.^61^ To better simulate cellular communication, direct co‐culture is necessary. Currently, most studies rely on 2D co‐cultures on polystyrene dishes. However, this culture approach may introduce confounding effects from physical cues and compromise cellular properties.^62^ Cell spheroids, on the other hand, closely replicate cell–cell and cell–matrix interactions seen in vivo and exhibit higher secretion levels of cytokines, growth factors, and chemokines compared to 2D cultured cells.^63^ The multicellular spheroid suspension co‐culture model emerged as the optimal choice for our study. While this approach eliminates the influence of other cell types by focusing on two specific cell types (fibroblasts and endothelial cells), it does not fully replicate the in vivo microenvironment.

To further elucidate the relationship between fibroblasts and endothelial cells in the in vivo state, we collected scar samples and normal skin samples from patients and performed scRNA‐seq analysis (Figure 5a). This analysis validated the findings of our previous investigations. Through our research and analysis, along with insights from other studies, we have recognized the close communication between fibroblasts and endothelial cells. The sequencing results have also highlighted certain genes and pathways that may serve as key intervention points. Therefore, delving deeper into the molecular mechanisms underlying the interaction between these two cell types and identifying targets for scar formation intervention may pave the way for future research endeavors.

## 5 CONCLUSION

This study highlights the crucial interplay between fibroblasts and endothelial cells, showcasing their ability to intricately regulate each other's behavior through paracrine secretion and cellular tight junctions. These findings shed light on the essential role of this communication in orchestrating the body's inflammatory response and facilitating wound healing and scar formation. By delving deeper into the molecular mechanisms underlying this interplay, we can unlock new avenues for the development of innovative therapeutic strategies, offering promising prospects for more effective treatment of tissue injuries and diseases.

## AUTHOR CONTRIBUTIONS

**Yaxin Tan:** Data curation (lead); formal analysis (lead); methodology (lead); writing – original draft (lead). **Mengde Zhang:** Data curation (lead); methodology (lead); software (lead). **Yi Kong:** Data curation (lead); formal analysis (lead); methodology (lead). **Fanliang Zhang:** Data curation (equal); validation (equal). **Yuzhen Wang:** Data curation (equal); methodology (equal); validation (equal). **Yuyan Huang:** Data curation (equal); software (equal). **Wei Song:** Data curation (equal); formal analysis (equal). **Zhao Li:** Supervision (equal); writing – original draft (equal). **Linhao Hou:** Data curation (equal). **Liting Liang:** Methodology (equal). **Xu Guo:** Data curation (supporting). **Qinghua Liu:** Methodology (supporting). **Chao Zhang:** Software (supporting). **Yu Feng:** Data curation (supporting). **Xiaobing Fu:** Conceptualization (lead); funding acquisition (lead); investigation (lead); resources (lead); supervision (lead). **Sha Huang:** Conceptualization (lead); funding acquisition (lead); investigation (lead); resources (lead); supervision (lead); writing – original draft (lead); writing – review and editing (lead).

## CONFLICT OF INTEREST STATEMENT

Any commercial or financial relationships that could be construed as a potential conflict of interest were absent of this research.

### PEER REVIEW

The peer review history for this article is available at https://www.webofscience.com/api/gateway/wos/peer‐review/10.1002/btm2.10630.

## Data Availability

The data that support the findings of this study are available from the corresponding author upon reasonable request.

## References

[1] Singer AJ , Clark RA . Cutaneous wound healing. N Engl J Med. 1999;341(10):738‐746. 10471461 10.1056/NEJM199909023411006

[2] Gurtner GC , Werner S , Barrandon Y , Longaker MT . Wound repair and regeneration. Nature. 2008;453(7193):314‐321. 18480812 10.1038/nature07039

[3] Rodrigues M , Kosaric N , Bonham CA , Gurtner GC . Wound healing: a cellular perspective. Physiol Rev. 2019;99(1):665‐706. 30475656 10.1152/physrev.00067.2017 PMC6442927

[4] Rinkevich Y , Walmsley GG , Hu MS , et al. Skin fibrosis. Identification and isolation of a dermal lineage with intrinsic fibrogenic potential. Science. 2015;348(6232):aaa2151. 25883361 10.1126/science.aaa2151 PMC5088503

[5] Plikus MV , Wang X , Sinha S , et al. Fibroblasts: origins, definitions, and functions in health and disease. Cell. 2021;184(15):3852‐3872. 34297930 10.1016/j.cell.2021.06.024 PMC8566693

[6] Griffin MF , Borrelli MR , Garcia JT , et al. JUN promotes hypertrophic skin scarring via CD36 in preclinical in vitro and in vivo models. Sci Transl Med. 2021;13(609):eabb3312. 34516825 10.1126/scitranslmed.abb3312 PMC8988368

[7] Henderson NC , Rieder F , Wynn TA . Fibrosis: from mechanisms to medicines. Nature. 2020;587(7835):555‐566. 33239795 10.1038/s41586-020-2938-9 PMC8034822

[8] Hinz B . Formation and function of the myofibroblast during tissue repair. J Invest Dermatol. 2007;127(3):526‐537. 17299435 10.1038/sj.jid.5700613

[9] Darby IA , Laverdet B , Bonté F , Desmoulière A . Fibroblasts and myofibroblasts in wound healing. Clin Cosmet Investig Dermatol. 2014;7:301‐311. 10.2147/CCID.S50046 PMC4226391 25395868

[10] Talbott HE , Mascharak S , Griffin M , Wan DC , Longaker MT . Wound healing, fibroblast heterogeneity, and fibrosis. Cell Stem Cell. 2022;29(8):1161‐1180. 35931028 10.1016/j.stem.2022.07.006 PMC9357250

[11] Hinz B . The extracellular matrix and transforming growth factor‐β1: tale of a strained relationship. Matrix Biol. 2015;47:54‐65. 25960420 10.1016/j.matbio.2015.05.006

[12] Zhu L , Liu L , Wang A , Liu J , Huang X , Zan T . Positive feedback loops between fibroblasts and the mechanical environment contribute to dermal fibrosis. Matrix Biol. 2023;121:1‐21. 37164179 10.1016/j.matbio.2023.05.001

[13] Ray S , Ju X , Sun H , Finnerty CC , Herndon DN , Brasier AR . The IL‐6 trans‐signaling‐STAT3 pathway mediates ECM and cellular proliferation in fibroblasts from hypertrophic scar. J Invest Dermatol. 2013;133(5):1212‐1220. 23303450 10.1038/jid.2012.499 PMC3626764

[14] Rafii S , Butler JM , Ding BS . Angiocrine functions of organ‐specific endothelial cells. Nature. 2016;529(7586):316‐325. 26791722 10.1038/nature17040 PMC4878406

[15] Huang C , Liu L , You Z , et al. Endothelial dysfunction and mechanobiology in pathological cutaneous scarring: lessons learned from soft tissue fibrosis. Br J Dermatol. 2017;177(5):1248‐1255. 28403507 10.1111/bjd.15576

[16] Korntner S , Lehner C , Gehwolf R , et al. Limiting angiogenesis to modulate scar formation. Adv Drug Deliv Rev. 2019;146:170‐189. 29501628 10.1016/j.addr.2018.02.010

[17] Chen Y , Ding BS . Comprehensive review of the vascular niche in regulating organ regeneration and fibrosis. Stem Cells Transl Med. 2022;11(11):1135‐1142. 36169406 10.1093/stcltm/szac070 PMC9672849

[18] Butler JM , Kobayashi H , Rafii S . Instructive role of the vascular niche in promoting tumour growth and tissue repair by angiocrine factors. Nat Rev Cancer. 2010;10(2):138‐146. 20094048 10.1038/nrc2791 PMC2944775

[19] Pasquier J , Ghiabi P , Chouchane L , Razzouk K , Rafii S , Rafii A . Angiocrine endothelium: from physiology to cancer. J Transl Med. 2020;18(1):52. 32014047 10.1186/s12967-020-02244-9 PMC6998193

[20] Ramasamy SK , Kusumbe AP , Adams RH . Regulation of tissue morphogenesis by endothelial cell‐derived signals. Trends Cell Biol. 2015;25(3):148‐157. 25529933 10.1016/j.tcb.2014.11.007 PMC4943524

[21] Woik N , Kroll J . Regulation of lung development and regeneration by the vascular system. Cell Mol Life Sci. 2015;72(14):2709‐2718. 25894695 10.1007/s00018-015-1907-1 PMC11113134

[22] Clark RA , Quinn JH , Winn HJ , Lanigan JM , Dellepella P , Colvin RB . Fibronectin is produced by blood vessels in response to injury. J Exp Med. 1982;156(2):646‐651. 7047672 10.1084/jem.156.2.646 PMC2186773

[23] Dalton CJ , Lemmon CA . Fibronectin: molecular structure, fibrillar structure and mechanochemical signaling. Cell. 2021;10(9):2443. 10.3390/cells10092443 PMC8471655 34572092

[24] Lipphardt M , Dihazi H , Jeon NL , et al. Dickkopf‐3 in aberrant endothelial secretome triggers renal fibroblast activation and endothelial‐mesenchymal transition. Nephrol Dial Transplant. 2019;34(1):49‐62. 29726981 10.1093/ndt/gfy100 PMC6322445

[25] Lian N , Li T . Growth factor pathways in hypertrophic scars: molecular pathogenesis and therapeutic implications. Biomed Pharmacother. 2016;84:42‐50. 27636511 10.1016/j.biopha.2016.09.010

[26] Wilgus TA . Vascular endothelial growth factor and cutaneous scarring. Adv Wound Care (New Rochelle). 2019;8(12):671‐678. 31750015 10.1089/wound.2018.0796 PMC6862968

[27] Tan Y , Suarez A , Garza M , Khan AA , Elisseeff J , Coon D . Human fibroblast‐macrophage tissue spheroids demonstrate ratio‐dependent fibrotic activity for in vitro fibrogenesis model development. Biomater Sci. 2020;8(7):1951‐1960. 32057054 10.1039/c9bm00900k PMC7179997

[28] Lim JTC , Kwang LG , Ho NCW , et al. Hepatocellular carcinoma organoid co‐cultures mimic angiocrine crosstalk to generate inflammatory tumor microenvironment. Biomaterials. 2022;284:121527. 35483200 10.1016/j.biomaterials.2022.121527

[29] Antoni D , Burckel H , Josset E , Noel G . Three‐dimensional cell culture: a breakthrough in vivo. Int J Mol Sci. 2015;16(3):5517‐5527. 25768338 10.3390/ijms16035517 PMC4394490

[30] Laschke MW , Menger MD . Life is 3D: boosting spheroid function for tissue engineering. Trends Biotechnol. 2017;35(2):133‐144. 27634310 10.1016/j.tibtech.2016.08.004

[31] Yao B , Wang R , Wang Y , et al. Biochemical and structural cues of 3D‐printed matrix synergistically direct MSC differentiation for functional sweat gland regeneration. Sci Adv. 2020;6(10):eaaz1094. 32181358 10.1126/sciadv.aaz1094 PMC7056319

[32] Zhang F , Zhang Z , Duan X , et al. Integrating zinc/silicon dual ions with 3D‐printed GelMA hydrogel promotes in situ hair follicle regeneration. Int J Bioprint. 2023;9(3):703. 37273992 10.18063/ijb.703 PMC10236330

[33] Duan X , Yuan X , Yao B , et al. The role of CTHRC1 in promotion of cutaneous wound healing. Signal Transduct Target Ther. 2022;7(1):183. 35701414 10.1038/s41392-022-01008-9 PMC9197944

[34] Mitrakas AG , Tsolou A , Didaskalou S , et al. Applications and advances of multicellular tumor spheroids: challenges in their development and analysis. Int J Mol Sci. 2023;24(8):6949. 37108113 10.3390/ijms24086949 PMC10138394

[35] Costa EC , Moreira AF , de Melo‐Diogo D , Gaspar VM , Carvalho MP , Correia IJ . 3D tumor spheroids: an overview on the tools and techniques used for their analysis. Biotechnol Adv. 2016;34(8):1427‐1441. 27845258 10.1016/j.biotechadv.2016.11.002

[36] He H , Suryawanshi H , Morozov P , et al. Single‐cell transcriptome analysis of human skin identifies novel fibroblast subpopulation and enrichment of immune subsets in atopic dermatitis. J Allergy Clin Immunol. 2020;145(6):1615‐1628. 32035984 10.1016/j.jaci.2020.01.042

[37] Liu X , Chen W , Zeng Q , et al. Single‐cell RNA‐sequencing reveals lineage‐specific regulatory changes of fibroblasts and vascular endothelial cells in keloids. J Invest Dermatol. 2022;142(1):124‐135.e11. 34242659 10.1016/j.jid.2021.06.010

[38] Efremova M , Vento‐Tormo M , Teichmann SA , Vento‐Tormo R . CellPhoneDB: inferring cell–cell communication from combined expression of multi‐subunit ligand–receptor complexes. Nat Protoc. 2020;15(4):1484‐1506. 32103204 10.1038/s41596-020-0292-x

[39] Aarabi S , Longaker MT , Gurtner GC . Hypertrophic scar formation following burns and trauma: new approaches to treatment. PLoS Med. 2007;4(9):e234. 17803351 10.1371/journal.pmed.0040234 PMC1961631

[40] Mony MP , Harmon KA , Hess R , Dorafshar AH , Shafikhani SH . An updated review of hypertrophic scarring. Cell. 2023;12(5):678. 10.3390/cells12050678 PMC10000648 36899815

[41] Wan B , Ganier C , Du‐Harpur X , et al. Applications and future directions for optical coherence tomography in dermatology. Br J Dermatol. 2021;184(6):1014‐1022. 32974943 10.1111/bjd.19553

[42] Aird WC . Endothelium as an organ system. Crit Care Med. 2004;32(5 Suppl):S271‐S279. 15118530 10.1097/01.ccm.0000129669.21649.40

[43] Ou L , Zhang P , Huang Z , et al. Targeting STING‐mediated pro‐inflammatory and pro‐fibrotic effects of alveolar macrophages and fibroblasts blunts silicosis caused by silica particles. J Hazard Mater. 2023;458:131907. 37379600 10.1016/j.jhazmat.2023.131907

[44] Veth TS , Francavilla C , Heck AJR , Altelaar M . Elucidating fibroblast growth factor‐induced kinome dynamics using targeted mass spectrometry and dynamic modeling. Mol Cell Proteomics. 2023;22:100594. 37328066 10.1016/j.mcpro.2023.100594 PMC10368922

[45] Yao L , Rathnakar BH , Kwon HR , et al. Temporal control of PDGFRα regulates the fibroblast‐to‐myofibroblast transition in wound healing. Cell Rep. 2022;40(7):111192. 35977484 10.1016/j.celrep.2022.111192 PMC9423027

[46] Zhang S , Tong X , Liu S , et al. AAV9‐Tspyl2 gene therapy retards bleomycin‐induced pulmonary fibrosis by modulating downstream TGF‐β signaling in mice. Cell Death Dis. 2023;14(6):389. 37391440 10.1038/s41419-023-05889-8 PMC10313802

[47] Chen W , Xia P , Wang H , et al. The endothelial tip‐stalk cell selection and shuffling during angiogenesis. J Cell Commun Signal. 2019;13(3):291‐301. 30903604 10.1007/s12079-019-00511-z PMC6732145

[48] Moya IM , Umans L , Maas E , et al. Stalk cell phenotype depends on integration of notch and Smad1/5 signaling cascades. Dev Cell. 2012;22(3):501‐514. 22364862 10.1016/j.devcel.2012.01.007 PMC4544746

[49] Bodnar RJ . Chemokine regulation of angiogenesis during wound healing. Adv Wound Care (New Rochelle). 2015;4(11):641‐650. 26543678 10.1089/wound.2014.0594 PMC4620517

[50] Lee J , Lee S , Kim SM , Shin H . Size‐controlled human adipose‐derived stem cell spheroids hybridized with single‐segmented nanofibers and their effect on viability and stem cell differentiation. Biomater Res. 2021;25(1):14. 33902733 10.1186/s40824-021-00215-9 PMC8074457

[51] Hu HH , Chen DQ , Wang YN , et al. New insights into TGF‐β/Smad signaling in tissue fibrosis. Chem Biol Interact. 2018;292:76‐83. 30017632 10.1016/j.cbi.2018.07.008

[52] Zhang YE . Non‐Smad signaling pathways of the TGF‐β family. Cold Spring Harb Perspect Biol. 2017;9(2):a022129. 27864313 10.1101/cshperspect.a022129 PMC5287080

[53] Fabre T , Kared H , Friedman SL , Shoukry NH . IL‐17A enhances the expression of profibrotic genes through upregulation of the TGF‐β receptor on hepatic stellate cells in a JNK‐dependent manner. J Immunol. 2014;193(8):3925‐3933. 25210118 10.4049/jimmunol.1400861 PMC4185218

[54] Park MJ , Moon SJ , Lee EJ , et al. IL‐1–IL‐17 signaling axis contributes to fibrosis and inflammation in two different murine models of systemic sclerosis. Front Immunol. 2018;9:1611. 30042768 10.3389/fimmu.2018.01611 PMC6048384

[55] Han M , Liu Z , Liu L , et al. Dual genetic tracing reveals a unique fibroblast subpopulation modulating cardiac fibrosis. Nat Genet. 2023;55(4):665‐678. 36959363 10.1038/s41588-023-01337-7

[56] Wei K , Korsunsky I , Marshall JL , et al. Notch signalling drives synovial fibroblast identity and arthritis pathology. Nature. 2020;582(7811):259‐264. 32499639 10.1038/s41586-020-2222-z PMC7841716

[57] Sun X , Nkennor B , Mastikhina O , Soon K , Nunes SS . Endothelium‐mediated contributions to fibrosis. Semin Cell Dev Biol. 2020;101:78‐86. 31791693 10.1016/j.semcdb.2019.10.015

[58] Altorok N , Wang Y , Kahaleh B . Endothelial dysfunction in systemic sclerosis. Curr Opin Rheumatol. 2014;26(6):615‐620. 25191994 10.1097/BOR.0000000000000112

[59] Leach HG , Chrobak I , Han R , Trojanowska M . Endothelial cells recruit macrophages and contribute to a fibrotic milieu in bleomycin lung injury. Am J Respir Cell Mol Biol. 2013;49(6):1093‐1101. 23885794 10.1165/rcmb.2013-0152OC PMC3931119

[60] Gunalan A , Mattos LS . Towards OCT‐guided endoscopic laser surgery‐a review. Diagnostics (Basel). 2023;13(4):677. 36832167 10.3390/diagnostics13040677 PMC9955820

[61] Borciani G , Montalbano G , Baldini N , Cerqueni G , Vitale‐Brovarone C , Ciapetti G . Co‐culture systems of osteoblasts and osteoclasts: simulating in vitro bone remodeling in regenerative approaches. Acta Biomater. 2020;108:22‐45. 32251782 10.1016/j.actbio.2020.03.043

[62] Randall MJ , Jüngel A , Rimann M , Wuertz‐Kozak K . Advances in the biofabrication of 3D skin *in vitro*: healthy and pathological models. Front Bioeng Biotechnol. 2018;6:154. 30430109 10.3389/fbioe.2018.00154 PMC6220074

[63] Liu D , Chen S , Win NM . A review of manufacturing capabilities of cell spheroid generation technologies and future development. Biotechnol Bioeng. 2021;118(2):542‐554. 33146407 10.1002/bit.27620

